# Biochemical characterization of the native α-carbonic anhydrase purified from the mantle of the Mediterranean mussel, *Mytilus galloprovincialis*

**DOI:** 10.1080/14756366.2017.1284069

**Published:** 2017-02-23

**Authors:** Rosa Perfetto, Sonia Del Prete, Daniela Vullo, Giovanni Sansone, Carmela Barone, Mosè Rossi, Claudiu T. Supuran, Clemente Capasso

**Affiliations:** a Istituto di Bioscienze e Biorisorse, CNR, Napoli, Italy;; b Dipartimento Neurofarba, Università degli Studi di Firenze, Sezione di Scienze Farmaceutiche, and Laboratorio di Chimica Bioinorganica, Polo Scientifico, Sesto Fiorentino, Florence, Italy;; c Dipartimento di Biologia, Università degli Studi di Napoli, Federico II, Napoli, Italy;; d Dipartimento di Agraria, Università degli Studi di Napoli, Federico II, Portici (Napoli), Italy

**Keywords:** Carbonic anhydrase, metalloenzymes, α-class enzyme, hydratase activity, mussel, bivalve, protonography

## Abstract

A α-carbonic anhydrase (CA, EC 4.2.1.1) has been purified and characterized biochemically from the mollusk *Mytilus galloprovincialis*. As in most mollusks, this α-CA is involved in the biomineralization processes leading to the precipitation of calcium carbonate in the mussel shell. The new enzyme had a molecular weight of 50 kDa, which is roughly two times higher than that of a monomeric α-class enzyme. Thus, *Mytilus galloprovincialis* α-CA is either a dimer, or similar to the *Tridacna gigas* CA described earlier, may have two different CA domains in its polypeptide chain. The *Mytilus galloprovincialis* α-CA sequence contained the three His residues acting as zinc ligands and the gate-keeper residues present in all α-CAs (Glu106-Thr199), but had a Lys in position 64 and not a His as proton shuttling residue, being thus similar to the human isoform hCA III. This probably explains the relatively low catalytic activity of *Mytilus galloprovincialis* α-CA, with the following kinetic parameters for the CO_2_ hydration reaction: k*_cat_* = 4.1 × 10^5^ s^−1^ and k*_cat_*/K*_m_* of 3.6 × 10^7^ M^−1^ × s^−1^. The enzyme activity was poorly inhibited by the sulfonamide acetazolamide, with a K*_I_* of 380 nM. This study is one of the few describing in detail the biochemical characterization of a molluskan CA and may be useful for understanding in detail the phylogeny of these enzymes, their role in biocalcification processes and their potential use in the biomimetic capture of the CO_2_.

## Introduction

Mollusk shell is composed of about 95% calcium carbonate (CaCO_3_) and 5% of organic components^1^. The shell is formed by three elements secreted by the mantle (the dorsal body of the animal): *a*) a thin outer periostracum; *b*) a middle prismatic, which is a crystalline form of calcium carbonate; *c*) an inner calcareous (nacreous) layer appearing with a vividness texture or iridescent mother-of-pearl (nacre), depending on the species^2–4^. Prismatic and nacreous layers are in the forms of calcium carbonate crystal; but, the two layers display a crystal polymorphism because the prismatic layer develops calcite and the nacreous layer forms aragonite^5^. The shell grows in circumference by the addition of material from the edge of the mantle, while grows in thickness by deposition from the general mantle surface. Calcium for shell growth is obtained from diet and/or from seawater, whereas carbonate is originated from the concentration of CO_2_/bicarbonate present in the tissue of the organism^6^. The interconversion of CO_2_ and HCO_3_^−^ is balanced naturally to maintain the equilibrium between dissolved inorganic carbon dioxide (CO_2_), carbonic acid (H_2_CO_3_), bicarbonate (HCO_3_^−^) and carbonate (CO_3_^2−^)^7–9^. The spontaneous hydration/dehydration reaction: CO_2 _+ H_2_O ⇋ HCO_3_^− ^+ H^+^ is very slow at physiological pH. In this context, a family of metalloenzymes, named carbonic anhydrase (CA; EC 4.2.1.1) assumes a pivotal role in the biomineralization process of the mollusks and is essential for the shell development^7^^,^^8^^,^^10–16^.

CAs, in fact, have the primary function to strongly accelerate the interconversion of CO_2_ and HCO_3_^−^ playing an important role during the calcium carbonate formation in mollusks, such as acid–base regulation, calcification and mineralization^17^^,^^18^. It has been reported in literature that intracellular or cytoplasmic membrane bound CAs are essential for cytoplasmic acid/base balance and for transport mechanisms of CO_2_ and carbonate ions; different CA isoforms has been identified in the calcifying epithelia of the mollusks^17^^,^^19^; CA activity has been reported in the extracellular skeletal matrix of the cnidarian exoskeleton, crustacean calcium storage concretions, fish otolith and molluskan shell^17^^,^^19^^,^^20^; moreover, nacrein, the protein involved in the nacreous layer formation of shell and pearl, identified for the first time in the Japanese pearl oyster *Pinctada fucata*, function has a CA because it has a CA domain in its amino acid sequence^21^.

The CA superfamily includes seven distinct classes known as the α, β, γ, δ, ζ, η and θ. In addition to biomineralization, these enzymes are involved in many physiologic processes, such as photosynthesis, respiration, CO_2_ transport, as well as metabolism of xenobiotics (e.g., cyanate in *Escherichia coli*)^13–15^^,^^22–31^. Some of the catalytically active α- and θ-CAs can also catalyze the hydrolysis of esters, such as 4-nitrophenyl acetate (4-NpA)^32^. However, no esterase activity was detected so far for enzymes belonging to the other five classes (β-, γ-, δ-, ζ- and η-CAs)^33^. The α-, β-, δ-, η- and, perhaps θ-CAs use Zn(II) ions at the active site, the γ-CAs are probably Fe(II) enzymes (but they are active also with bound Zn(II) or Co(II) ions)^34–41^, whereas the ζ-class CAs are cambialistic enzymes, active both with Cd(II) or Zn(II) bounded within the active site in order to perform the physiologic reaction catalysis^42–44^. The metal ion from the enzyme active site is coordinated by three His residues in the α-, γ-, δ- and θ-classes, by one His and two Cys residues in β- and ζ-CAs or by two His and one Gln residues in η-class with the fourth ligand being a water molecule/hydroxide ion acting as nucleophile in the catalytic cycle of the enzyme^8^^,^^12^^,^^13^^,^^45–47^. In metazoans, CAs belong predominantly to the α-CA family, but recently β-CA family members have been identified^17^. In the last years, CAs have acquired a great importance in biotechnological applications, such as in the achievement of an artificial respiration system, selective biosensors for metal ions, and in the carbon capture process (CCP)^48^^,^^49^. In the CCP context, a number of CO_2_ sequestration methods have been proposed in order to capture CO_2_ using different types of CA enzymatic bioreactors^48^^,^^50^.

In the present study, we characterized and determined the kinetic constants of the CA purified from the mantle tissue of the bivalve Mediterranean mussel, *Mytilus galloprovincialis*. This CA has been purified by ammonium sulfate precipitation and ion-exchange chromatography followed by affinity chromatography. From the determination of its *N*-amino terminal sequence the purified metalloenzyme it has been assigned to the α-class of the CA superfamily. The kinetic study of the active α-CA in the mantle of the adult specimens of *Mytilus galloprovincialis* may provide further information on the physiological role and function of this metalloenzymes in the process of biomineralization of the bivalves. This is one of the few contributions, apart the characterization of the α-CA from *Tridacna gigas*, in which molluskan CAs are investigated in detail at the biochemical level^51^.

## Materials and methods

### Chemicals

All the chemicals were commercial products of the purest quality and purchased from Sigma. Immobilion-P membranes were from Perkin-Elmer (Waltham, MA).

### Animals

Adult specimens of *Mytilus galloprovincialis* were collected in the proximity of Gulf of Naples. The mussels were maintained in seawater at a temperature of 4 °C. Bivalve mantels were quickly removed and frozen at −70 °C.

### Enzyme purification

All the purification steps were carried out at a temperature of 4 °C. Approximately, 20 g of mussel mantles were homogenized in 50 ml of 20 mM Tris-HCl buffer pH 8.3 containing 10^−3^ M PMSF, 10^−3^ M benzamidine and 2 × 10^−3^ M EDTA. The homogenate was centrifuged twice for 30 min at 12,000 × *g*, and the resulting supernatant was centrifuged again for 45 min at 100,000 × *g*. This supernatant containing approximately 200 mg protein in a volume of 60 ml was subject to an ammonium sulfate precipitation. An amount of ammonium sulfate gradually was added to obtain saturations of 30%. After resting for 14 h at 4 °C, the sample was centrifuged at 12,000 × *g* at 4 °C for 30 min. The hydratase activity was detected in the supernatant fraction. This fraction was dialyzed against 20 mM Tris-HCl buffer pH 8.3. An amount of samples containing 5 mg of total protein was applied to a 4.6 × 50 mm anion-exchange Q column (BioSuite Q-PEEK, Waters Corporation) mounted on an Ultimate 3000 HPLC system (Dionex). The column was pre-equilibrated with 20 mM Tris-HCl, pH 7.5 (Buffer A). The CA was eluted by a linear gradient from 0% Buffer A to 100% Buffer B (20 mM Tris-HCl, pH 7.5, containing 0.5 M NaCl) with a flow rate set to 1 ml/min with a continuous monitoring of the absorbance at 280 and 220 nm; 1-ml fractions were collected. The fractions were tested for the CA presence using the protonography technique. A peak of CA activity was eluted at a concentration of NaCl of approximately 0.1 M. At this stage of the purification, the CA was 60% pure and the obtained recovery was of 1 mg of the native protein. The active fraction was pooled, dialyzed against 20 mM Tris-HCl buffer pH 7.5 and further purified by affinity chromatography on a p-aminomethylbenzenesulfonamide agarose resin (pAMBS; Sigma-Aldrich). 1 ml of pAMBS resin was applied to an empty econocolumn (BioRad) and equilibrated with 0.1 M Tris-HCl, pH 7.5 buffer containing 0.2 M K_2_SO_4_, 0.5 mM EDTA. The sample containing about 1 mg of total protein was loaded on the p-AMBS column equilibrated as aforementioned. Unbound proteins were removed by washing extensively with the same buffer. The bound CA was eluted using 0.4 M KSCN dissolved in 0.1 M Tris-HCl, pH 7.5 buffer. The CA-containing fractions were pooled, dialyzed and concentrated by ultrafiltration. The CA-containing sample was subject to protonography. At this stage of the purification, the CA isolated from *M. galloprovincialis* was 90% pure with a total concentration of 0.1 mg. Its N-amino terminal sequence was determined for assigning the CA at one of the seven classes of CA described in literature.

### SDS-PAGE, electroblotting and sequencing

Sodium dodecyl sulfate (SDS)-polyacrylamide gel electrophoresis (PAGE) was carried out according to Laemmli^52^. Samples were dissolved in buffer with 5% b-mercaptoethanol. Gel was stained with Coomassie blue. Blotting from gel onto an Immobilion-P membrane was performed as described by Matsudaira^53^. N-terminal sequencing was performed on the blotted protein by automated Edman degradation^54^. Protein concentration was determined by Bio-Rad assay kit.

### Carbonic anhydrase assay

CA activity assay was a medication of the procedure described by Capasso et al.^55^. Briefly, the assay was performed at 0 °C using CO_2_ as substrate following the pH variation due to the catalyzed conversion of CO_2_ to bicarbonate. Bromothymol blue was used as the indicator of pH variation. The production of hydrogen ions during the CO_2_ hydration reaction lowers the pH of the solution until the color transition point of the dye is reached. The time required for the color change is inversely related to the quantity of CA present in the sample. Wilbur-Anderson units were calculated according to the following definition: One Wilbur-Anderson unit (WAU) of activity is defined as (T0 − T)/T, where T0 (uncatalyzed reaction) and T (catalyzed reaction) are recorded as the time (in seconds) required for the pH to drop from 8.3 to the transition point of the dye in a control buffer and in the presence of enzyme, respectively.

### Esterase activity

Activity for p-nitrophenylacetate (p-NpA) hydrolysis was determined at 0 °C using a modification of the method proposed by Armstrong et al.^56^. The reaction mixture contained 0.3 ml of freshly prepared 3 mM p-NpA and 0.7 ml of 15 mM Tris sulfate buffer, pH 7.6. An aliquot of enzyme solution was added, and the catalyzed reaction was monitored reading the increase in A_348_ nm for 5 min. The catalyzed reactions were corrected for the non-enzymatic reaction. One enzyme unit was defined as the amount capable of producing an OD_348 nm_ = 0.03 in 5 min.

### Kinetic and inhibition assays

An applied photophysics stopped-flow instrument has been used for assaying the CA catalyzed CO_2_ hydration activity. Phenol red (at a concentration of 0.2 mM) has been used as indicator, working at the absorbance maximum of 557 nm, with 20 mM Tris (pH 8.3) as buffer, and 20 mM NaClO_4_ (for maintaining constant the ionic strength), following the initial rates of the CA-catalyzed CO_2_ hydration reaction for a period of 10–100 s. The CO_2_ concentrations ranged from 1.7 to 17 mM for the determination of the kinetic parameters (by Lineweaver–Burk plots) and inhibition constants. For each inhibitor at least six traces of the initial 5–10% of the reaction have been used for determining the initial velocity. The uncatalyzed rates were determined in the same manner and subtracted from the total observed rates. Stock solutions of inhibitor (10–100 mM) were prepared in distilled–deionized water and dilutions up to 0.01 mM were done thereafter with the assay buffer. Inhibitor and enzyme solutions were preincubated together for 15 min at room temperature prior to assay, in order to allow for the formation of the E–I complex or for the eventual active site mediated hydrolysis of the inhibitor. The inhibition constants were obtained by non-linear least-squares methods using PRISM 3 and the Cheng–Prusoff equation, as reported earlier, and represent the mean from at least three different determinations.

### Protonography

Protonography is a simple and inexpensive method, similar to zymography, which allowed the detection of CA activity on the polyacrylamide gel following the formation of H^+^ ions produced by hydratase activity of CAs^57–59^. Samples were run on SDS-PAGE at a concentration of about 1 μg per well. SDS-Page was carried out as described in the section “SDS-PAGE”, with the exception that samples were mixed with Laemmli loading buffer without 2-mercaptoethanol and without boiling the samples, in order to prevent protein denaturation induced by heating. The gel was run at 180 V until the dye front ran off the gel^57–59^. Following the electrophoresis, the gel was treated with Triton X-100 at 2.5% and kept under stirring for one hour to remove the SDS. The gel was subjected to a washing step of 20 min with 100 mM Tris-HCl, pH 8.3, containing 10% isopropanol. It was washed two times for 10 min. Finally, the gel was incubated for 30 min at 4 °C under stirring with 0.1% bromothymol blue (BTB, the pH indicator) in 100 mM Tris-HCl pH 8.3. To detect the hydratase activity, the gel was immersed in distilled water saturated with CO_2_ prepared by bubbling CO_2_ into 200 ml of distilled water for about 3 h. The localized decrease of the pH value, due to the presence of the enzymatic activity of CAs, was detected through the formation of yellow band due to the change of color of the BTB from blue (alkaline pH) to yellow (acidic pH)^29^^,^^57–61^.

## Results and discussion

### Purification of the native form of CA

**Figure 1.**
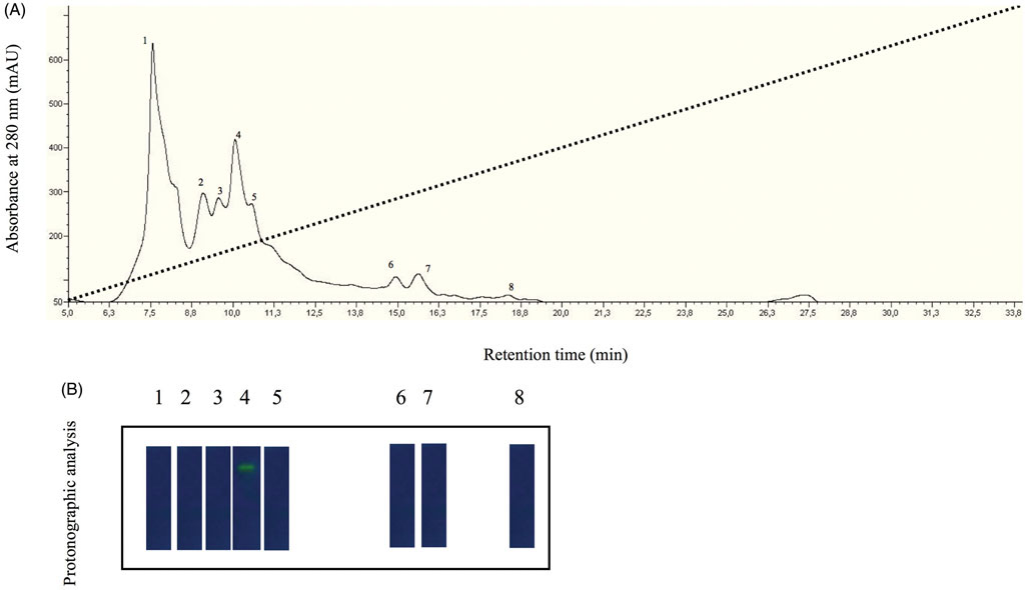
(A) Elution profile from the anion-exchange chromatography column of the supernatant obtained from the ammonium sulfate precipitation. Dot line represents the linear gradient from 0 to 0.5 NaCl; (B) Protonographic analysis carried out on the peaks eluted from the column. The yellow band denotes the hydratase activity due to the native CA purified by the mantles of the Mediterranean mussels, *M. galloprovincialis*.

The native CA was isolated and purified to homogeneity at 4 °C from the mantles of about 30 mussels belonging to the species *Mytilus galloprovincialis*, the Mediterranean mussel. Most of the CA activity was recovered in the soluble fraction of cell extract after ultracentrifugation as described in “Materials and methods” section. Contaminants proteins, such as actin and chitinase, were precipitated by addition of solid ammonium sulfate (30% saturation). Following the ammonium sulfate precipitation, the hydratase activity was detected in the supernatant fraction, which was fractioned by anion-exchange chromatography as showed in Figure 1. The elution profile from anion-exchange chromatography showed the presence of at least 8 peaks with a significant absorption at 280 nm (Figure 1, panel A). All the peaks were analyzed by protonographic technique, which allowed the detection of CA activity on the polyacrylamide gel following the formation of H^+^ ions produced by hydratase activity of CAs. The protonogram was stained with bromothymol blue, an indicator monitoring the pH variation. This dye appears blue in its deprotonated form, while its color changes to yellow in the protonated form. The production of hydrogen ions during the CO_2_ hydration lowers the pH of the solution until the transition point of the dye is reached (pH 6.8) and the yellow color appears on the gel (Figure 1, panel B). As shown in Figure 1, the protonograms clearly showed a hydratase activity only associated to the peak number 4 eluted at a concentration of NaCl of approximately 0.1 M and with a retention time of about 10 min. At this stage of the purification, the CA was 60% pure as in agreement with the SDS-PAGE analysis of the native enzyme (data not shown). The hydratase-containing peak was further purified by pAMBS affinity chromatography.

### SDS-Page and protonography

**Figure 2.**
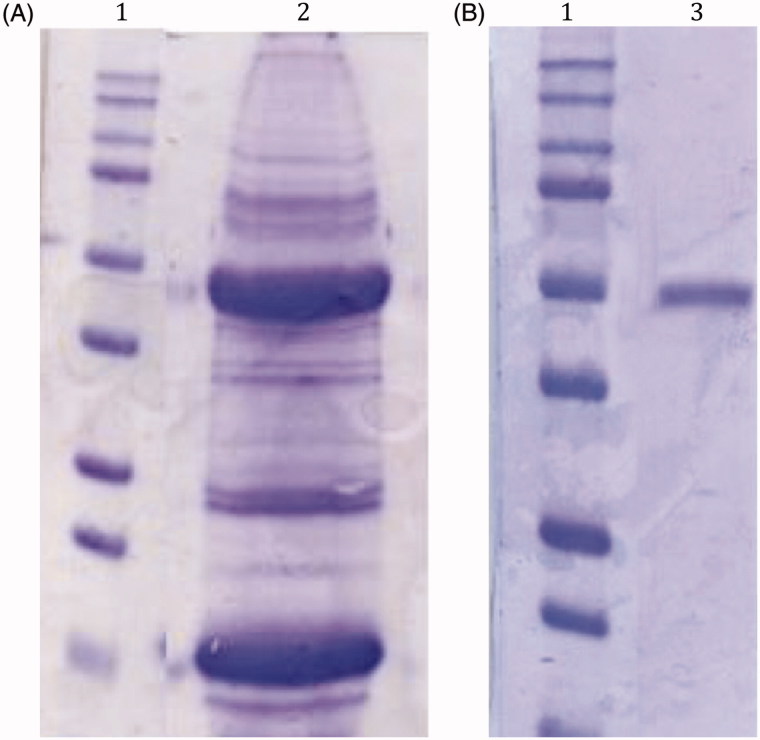
SDS-Page of the native CA purified from the mantles of *M. galloprovincialis*. Panel A, lane 2: cell extract protein from the mussel mantles before the purification; Panel B, lane 3: purified CA from pAMBS affinity column. Panel A and B, lane 1: molecular markers. Starting from the top: 250 kDa, 150 kDa, 100 kDa, 75 kDa, 50 kDa, 37 kDa, 25 kDa, 20 kDa, 15 kDa and 10 kDa.

In Figure 2 is shown the SDS-PAGEs obtained analyzing the sample after the tissue homogenization of the mussels (panel A) and the column affinity chromatography (panel B). The native CA was purified to an apparent homogeneity, as indicated by a single protein band after SDS-PAGE (Figure 1, panel B, lane 3). The molecular weight estimated by SDS-PAGE was 50.0 kDA under both reducing and non-reducing conditions. Generally, a subunit molecular mass of about 26.0 kDa was calculated on the basis of the amino acid sequence translated from the α- or β-CAs usually found in metazoans, although the *Tridacna gigas* CA was observed to possess a MW of 70 kDA, being a dual glycoprotein with two different CA domain, one at the amino-, the other at the carboxy-terminal parts, of the protein^51^. So, with the information available at this moment, we cannot rule out that the *M. galloprovincialis* CA may be a homodimer, or as the *T. gigas* enzyme, a glycoprotein with two different CA domains.

**Figure 3.**
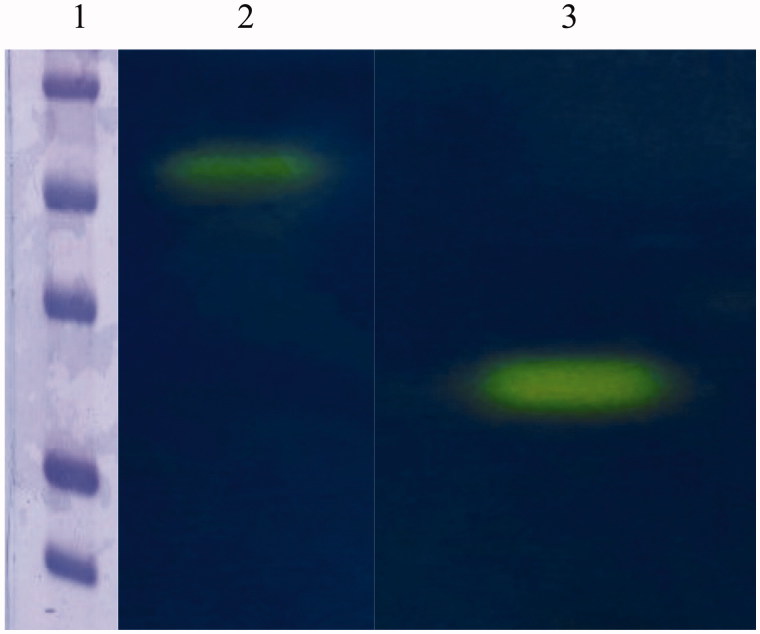
Protonogram obtained using the native CA from *Mytilus galloprovincialis* and the commercial bovine CA (bCA). Lane 2: mussel CA protonogram showing a band (yellow color) with an apparent molecular weight of 50 kDa; Lane 1: bovine CA is present in a monomeric state corresponding at a molecular weight of about 26 kDa; Lane 1: molecular markers. Starting from the top: 75 kDa, 50 kDa, 37 kDa, 25 kDa and 20 kDa.

The hydratase activity of the purified mussel CA was confirmed by protonography. Figure 3 showed the protonogram obtained with the native mussel CA. The commercial bovine CA (bCA) was used as positive control. The yellow band corresponded to the CA position on the gel. The SDS-PAGE and protonogram showed clearly that the native mussel CA had a dimeric arrangement, suggesting that this enzyme may acts as a physiological dimer. This is very intriguing because generally α-CAs are monomeric enzymes. But, in the last years, it has been discovered dimeric arrangements for several members of the α-class, such as SspCA, from *Sulfurihydrogenibium yellowstonense* YO3AOP1^39^; NgCA, from *Neisseria gonorrhoeae*^62^; CrCA, from *Chlamydomonas reinhardtii*^63^; AoCA, from *Aspergillus oryzae*^64^; and three human isoforms, indicated with the acronyms hCA VI, hCA IX and hCA XII^65–67^. Moreover, CAs (α-type) from mammalian sources catalyze the reversible hydrolysis of esters. Thus, with p-NpA as substrate, the presence of esterase activity was investigated in the native mussel enzyme. The mussel CA showed an esterase specific activity 100 times smaller than that of the commercially available bovine bCA II.

### Primary structure analysis

**Figure 4.**
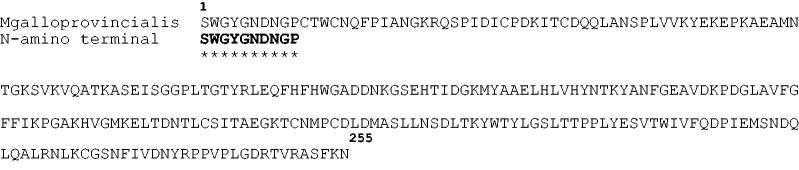
Amino acid sequence of CA from *Mytilus galloprovincialis* deposited in the protein data bank. In bold, the N-amino terminal sequence obtained from the electroblotted mussel CA. The asterisk indicates amino acid identity.

**Figure 5.**
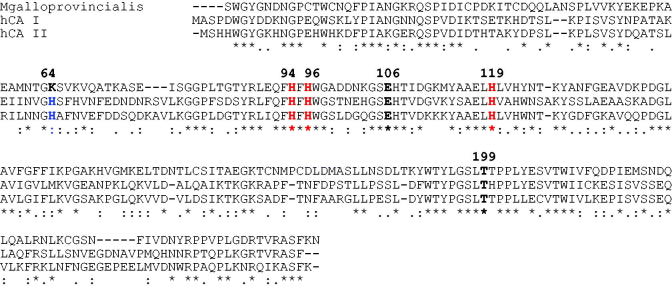
Amino acid multialignment obtained using the *Mytilus galloprovincialis* CA and *Homo sapiens* CA isoforms (hCA I and hCA II). The zinc ligands (His94, 96 and 119) and the gate-keeper residues (Glu106 and Thr199) are conserved in the mussel and mammalian sequences. The proton shuttle residue (His64) is missing in the *M. galloprovincialis* enzyme. hCA I numbering system was used. The asterisk (*) indicates identity at all aligned positions; the symbol (:) relates to conserved substitutions, while (.) means that semi-conserved substitutions are observed. Multialignment was performed with the program Muscle, version 3.1.

The N-amino terminal sequence of the electroblotted enzyme was found to be SWGYGNDNGP. Using the Basic Local Alignment Search Tool (BLAST) and using as query sequence the N-amino terminal obtained as described above, the purified native CA completely matched (query cover 100%) the N-amino terminal of the α-CA previously identified in the genome of the Mediterranean mussel, *Mytilus galloprovincialis* (Figure 4). The *M. galloprovincialis* CA is a polypeptide chain formed by 255 amino acid residues. In Figure 5, the *M. galloprovincialis* CA sequence was aligned with *Homo sapiens* CA isoforms (hCA I and hCA II) sequences. It may be observed that similar to the other investigated α-CA, the mussel metalloenzyme has the conserved three His ligands, which coordinate the Zn(II) ion crucial for catalysis (His94, 96 and 119, hCA I numbering system). Interesting to note that the proton shuttle residue (His64) is conserved in the human isoforms, but is missing in the mussel enzyme, being replaced by a Lys residue. This is also the situation for the human isoform hCA III, which has Lys64 instead of His64, and also shows a reduced catalytic activity compared with isoforms hCA I and II^68^. This residue assists the rate determining step of the catalytic cycle transferring a proton from the water coordinated to the Zn(II) ion to the environment, with formation of zinc hydroxide representing the nucleophilic species of the enzyme. A Lys in position 64 is less efficient than a His for assisting this process, which explains why the molluskan enzyme has a lower catalytic activity compared with hCA I and II^68^. *M. galloprovincialis* enzyme also has the gate-keeping residues (Glu106 and Thr199), which orientate the substrate for catalysis, and are also involved in the binding of inhibitors.

## Enzyme kinetics

Using the stopped-flow technique, the kinetic parameters were determined for the native α-CA using CO_2_ as a substrate^69^. The activity of the mussel enzyme was compared with that of other α-CAs such as *Homo sapiens* isoforms (hCA I and hCA II), *Stylophora pistillata* isoforms (STPCA and STPCA-2) (Table 1)^18^^,^^70^^,^^71^. As shown in Table 1, the mussel α-CA had a k*_cat_* = 4.1 × 10^5^ s^−1^ with a k*_cat_*/K*_m_* ratio 1.38 times lower with respect to that of the hCA I, which possessed a k*_cat_* of 2.0 × 10^5^ s^−1^. Moreover, the kinetic constants of the native mussel CA are very similar to those obtained for the coral isoform STPC and only slightly lower than the isoform STPCA-2. This is very intriguing since mussel α-CA respect to the two coral isoforms, lacked of a His64. This residue is involved in the transfer of proton from the water coordinated to the Zn(II) ion to the environment with the function to accelerate the rate of the catalytic cycle. STPCA-2 from scleractinian coral *Stylophora pistillata* is considered highly active and has an intracellular localization in the oral endoderm and in the aboral tissue of the coral. Interesting to note that the native mussel CA is not well affected by acetazolamide inhibition showing a K*_I_*of 380 mM (Table 1). The intracellular localization of the native mussel CA and its kinetic similarity with STPCA-2, could allow that the *Mytilus galloprovincialis* α-CA is involved in pH regulation and/or in the delivery of bicarbonate helping the mussel during the formation of the shell.

**Table 1.** Kinetic parameters for the CO_2_ hydration reaction catalyzed by the purified native mussel CA, the *Homo sapiens* CA isoforms (hCA I and hCA II) and coral CA isoforms (STPCA and STPCA-2). Acetazolamide (AAZ) inhibition data are also shown.

Enzyme	Class	k_*cat*_ (s^−1^)	k_*cat*_/K_*m*_ (M^−1^ × s^−1^)	K_*I*_ (acetazolamide) (nM)
hCA I	α	2.0 × 10^5^	5.0 × 10^7^	250
hCA II	α	1.4 × 10^6^	1.5 × 10^8^	12
STPCA	α	3.1 × 10^5^	4.6 × 10^7^	16
STPCA-2	α	5.6 × 10^5^	8.3 × 10^7^	74
*M. galloprovincialis*	α	4.1 × 10^5^	3.6 × 10^7^	380

Errors in the range of ±5% of the reported data from three different assays.

## Conclusions

A α-CA has been purified and characterized biochemically from the mollusk *Mytilus galloprovincialis*. As in most mollusks, this α-CA seems to be involved in the biomineralization processes leading to the precipitation of calcium carbonate in the mussel shell. The new enzyme has a molecular weight of 50 kDa, which is roughly two times higher than that of a monomeric α-class enzyme. Thus, *Mytilus galloprovincialis* α-CA is either a dimer, or similar to the *Tridacna gigas* CA described earlier, may have two different CA domains in its polypeptide chain. The *Mytilus galloprovincialis* α-CA sequence contains the three His residues acting as zinc ligands and the gate-keeper residues (Glu106-Thr199) present in all α-CAs, but had a Lys in position 64 and not a His as proton shuttling residue, being thus similar to the human isoform hCA III. This probably explains the relatively low catalytic activity of *Mytilus galloprovincialis* α-CA, with the following kinetic parameters for the CO_2_ hydration reaction: k*_cat_*= 4.1 × 10^5^ s^−1^ and k*_cat_*/K*_m_* of 3.6 × 10^7^ M^−1^ × s^−1^. The enzyme activity was poorly inhibited by the sulfonamide acetazolamide, with a K*_I_* of 380 nM. This study is one of the few describing in detail the biochemical characterization of a molluskan CA and may be useful for understanding in better detail the phylogeny of these enzymes and their role in biocalcification processes. In addition, the mussel CA is an attractive candidate for its potential use in carbon dioxide sequestration. The recombinant DNA technology will make possible the ingegnerization of the mussel CA and production of large quantities of the metalloenzyme with the aim to use the *M. galloprovincialis* CA, either free or immobilized, in the CO_2_ biomimetic capture process.
